# A “Single-Use” Ceramic-Based Electrochemical Sensor Chip Using Molecularly Imprinted Carbon Paste Electrode

**DOI:** 10.3390/s20205847

**Published:** 2020-10-16

**Authors:** Aaryashree, Yuuto Takeda, Momoe Kanai, Akihiko Hatano, Yasuo Yoshimi, Masahito Kida

**Affiliations:** 1 Department of Applied Chemistry, Shibaura Institute of Technology, Tokyo 135-8548, Japan; aaryashree.c7@sic.shibaura-it.ac.jp (A.); mc19009@shibaura-it.ac.jp (Y.T.); mc20012@shibaura-it.ac.jp (M.K.); 2 Department of Chemistry, Shibaura Institute of Technology, Saitama 337-8570, Japan; a-hatano@sic.shibaura-it.ac.jp; 3 R&D Center, Technology Development Division, NGK Spark Plug, Co., Komaki 485-8510, Japan; m-kida@mg.ngkntk.co.jp

**Keywords:** electrochemical sensor, molecularly imprinted polymer, disposable sensor, therapeutic drug monitoring, ceramic chip

## Abstract

An inexpensive disposable electrochemical drug sensor for the detection of drugs (vancomycin, meropenem, theophylline, and phenobarbital) is described. Molecularly imprinted polymer (MIP) templated with the target drugs was immobilized on the surface of graphite particles using a simple radical polymerization method and packed into the working electrode of a three-electrode ceramic-based chip sensor. Differential pulse voltammetry (DPV) was used to determine the relationship between the response current and the concentration of the targeted drug while using one sensor chip for one single operation. The time required for each DPV measurement was less than 2 min. Concentrations corresponding to the therapeutic range of these drugs in plasma were taken into account while performing DPV. In all the cases, the single-used MIP sensor showed higher sensitivity and linearity than non-imprinted polymer. The selectivity test in drugs with a structure similar to that of the target drugs was performed, and it was found that MIP-based sensors were more selective than the untreated ones. Additionally, the test in whole blood showed that the presence of interfering species had an insignificant effect on the diagnostic responses of the sensor. These results demonstrate that the disposable MIP-sensor is promising for quick and straightforward therapeutic drug monitoring to prevent the toxic side effects and the insufficient therapeutic effect due to the overdose and underdose, respectively.

## 1. Introduction

In general, most of the mainstream drugs are administered in a very standard way of unit per body mass basis, with adjustments based on the overall response as empirically assessed by the physician. For a defined group of drugs, the pharmacodynamics is evaluated accurately through simple laboratory measurements. However, the pharmacokinetics–pharmacodynamic relationship among specific subsets of drugs is inconsistent, and the pharmacokinetics in such drugs is often complicated, rendering the drug doses and control quite challenging. Therefore, for such drugs, therapeutic drug monitoring (TDM) is an incredibly useful technique [1].

TDM is the clinical method of calculating particular medications at prescribed intervals to maintain a steady concentration in the bloodstream of a patient and improve individual dosage regimens. The use of TDM for most medications is undesirable and therefore it is primarily used for tracking drugs: (a) with limited therapeutic ranges, (b) with significant pharmacokinetic variation, (c) for which target doses are difficult to control, and (d) known to cause therapeutic and adverse effects. However, TDM is yet to be widely used, especially in developing countries, due to the required blood-level analysis methods [2,3]. Including immunoassay or liquid chromatography with tandem mass spectroscopy (LC-MS/MS), these blood-level assessments remain prohibitively costly (approximately USD 60–200 per sample per person), and as a result, are yet to gain significant social traction, due to fundamental engineering challenges, in those communities most in need and most at risk. The development of facile, inexpensive, and highly functional sensors for multiple drug assessment, directly in the blood, which can be used in developing countries, is urgently required to overcome such a societal paradox. Most of these drugs are used for treating chronic conditions such as epilepsy, chronic asthma, intra-abdominal infections, and some severe bacterial infections [4,5,6,7,8,9,10]. Still, certain toxic antibiotics, such as vancomycin and meropenem, require TDM if used for more than two days [11,12,13,14].

Therefore, an inexpensive disposable sensor that can determine the drug concentration in blood with a simple operation is required. In this study, we developed a ceramic-based sensor chip using a carbon paste electrode grafted with a molecularly imprinted polymer (MIP) as the molecular-recognition element for various drugs. A MIP is obtained by copolymerization of a cross-linking monomer and functional monomer, which has an affinity with the target molecule in the presence of the target molecule as a template [15,16]. The use of MIPs is one of the most generic, versatile, scalable, and cost-effective approaches in creating synthetic molecular receptors [17,18,19,20]. The significant advantageous properties contributing to the growing interest in MIPs are their high affinity and selectivity, stability, the simple preparation, and ease of integration into practical applications [21,22,23]. Primarily, MIP can be obtained economically by a simple procedure.

Nevertheless, MIPs are hardly used in reality as the methodology for reading out the specific binding event by MIPs is yet to be established. We have developed a sensing method using electrodes grafted with MIPs [17,24,25,26,27,28]. The sensor using MIP-grafted electrodes responds to the target very quickly (within several tens of seconds). However, MIP is grafted through radical polymerization, whose precise control is difficult; thus, poor reproducibility of electrodes had been a problem. Furthermore, since a clinical sensor has to be disposed of after every sample measurement, considering the hygiene, a high uniformity among the sensors is required. Recently, we developed a carbon paste electrode with MIP grafted directly onto the graphite particles [17]. Uniform electrodes can be obtained by thorough mixing of the MIP-grafted graphite and oil and dispensed onto the base of the sensor and can work as disposable sensors.

In this work, we developed sensor chips by dispensing carbon paste grafted with MIP on a ceramic chip. The feasibility of the sensor chip as a disposable sensor for drugs that require TDM: antibacterial drugs (vancomycin and meropenem), bronchodilators (theophylline), and antiepileptic drugs (phenobarbital), was evaluated by differential pulse voltammetry in saline or whole blood samples. The main highlights of the presented sensor are: (a) easy to use, (b) single-use or disposable, (c) measurement in whole blood, (d) only 50 μL of solution required for sensing, (e) reagentless measurement technique, (f) faster TDM than immunoassay and liquid chromatography, and (g) low cost, so that it can be used in the developing countries

## 2. Materials and Methods

### 2.1. Materials

Methacrylic acid (MAA), N, N’-methylenebisacrylamide (MBAA) and acrylamide (AAm), ethylene glycol dimethacrylate (EDMA), vancomycin hydrochloride, phenobarbital sodium salt, phenytoin, caffeine anhydrous, theophylline, meropenem trihydrate, water-soluble carbodiimide (WSC), anhydrous sodium sulphate and sodium chloride were bought from Wako Pure Chemical Industry (Osaka, Japan). Methanol and N, N-dimethylformamide (DMF) were purchased from Kanto Chemical Co., Ltd. (Tokyo, Japan). The 3-Ferrocenoylpropyonic acid and allylamine were purchased from Tokyo Chemical Industry (Tokyo, Japan). Imipenem monohydrate was obtained from Combi-blocks (San Diego, CA, USA), and teicoplanin was obtained from Biodivision (Tokyo, Japan). Bovine blood for testing was bought from the Tokyo Shibaura Zoki Corporation (Tokyo, Japan) (5 g/L sodium citrate was added into the blood as an anticoagulant.) MAA and EDMA were used after purification by reduced-pressure distillation. Spherical graphite particles, 8 µm in diameter (SG-BH8), were donated by Ito Graphite Co., Ltd. (Kuwana, Japan).

### 2.2. Preparation of Allylaminocarboxy Propionic-3-Ferrocene (ACPF)

**Figure 1:**
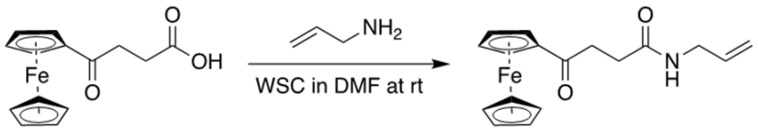
Preparation of the redox monomer allylaminocarboxy propionic-3-ferrocene (ACPF).

ACPF was synthesized as a functional monomer for vancomycin. The 3-Ferrocenoylpropyonic acid (0.1 mol, 28.6 g) was added to a solution of WSC (0.11 mmol, 21.6 g) and allylamine (0.12 mol, 6.85 g, 9.02 mL) in 200 mL of DMF. The reaction mixture was stirred at room temperature for two hours under argon. Most of the DMF was evaporated under reduced pressure. The residue was dissolved in 120 mL of ethylacetate (AcOEt), washed twice with 120 mL of water, and then dried over anhydrous sodium sulphate. After filtration, the solvent was removed with a rotary evaporator. The crude material was purified via silica gel column chromatography using *n*-hexane: AcOEt (3:2) to afford N-Allyl 4-ferrocene-4-oxopropylamide (AFPA) as a brown syrup (19.1 g, 59%). ^1^H NMR (CDCl_3_): δ 1.65 (2H, s), 2.59 (2H, *J* = 6.6 Hz, t), 3.14 (2H, *J* = 6.4 Hz, t), 3.91 (2H, *m*), 4.24 (5H, s), 4.52 (2H, m), 4.81 (1H, *J* = 2.0 Hz, d), 5.22 (2H, *J* = 13.6, 27.2 Hz, dd); 5.85 (1H, m), 5.98 (1H, br); ^13^C NMR (CDCl_3_): ESI-HRMS calculated for C_34_H_38_Fe_2_N_2_NaO_4_: 2673.1403; found 2673.1428. ACPF was then produced by the coupling reaction of AFPA in the DMF solution at room temperature, as shown in Figure 1.

### 2.3. Preparation of MIPs Grafted on Graphite Particles

Initiator-introduced graphite (IG) for the radical polymerization was prepared by adding a diethyldithiocarbamate methylene group onto the surface of the graphite particle through chloromethylation, following a previously reported method [17]. The MIP for all the drugs grafted on the surface of the IG was prepared using a general radical polymerization process in the fluidized bed of IG in the solution of monomers and templates. The detailed preparation of the MIP for the individual drugs is as below:

#### 2.3.1. Vancomycin MIP

The functional monomer MAA (0.12 mol, 1.08 g), the cross-linking monomer MBAA (0.02 mol, 3.08 g) the cross-linking regulator AAm (0.015 mol, 1.08 g), redox monomer ACPF (0.44 mmol, 0.3 g) and the template vancomycin hydrochloride (0.24 mmol, 0.36 g) were dissolved in a mixed solvent of 4 mL distilled water and 18 mL DMF. The solution and 0.5 g IG were placed in a quartz crystal test tube (26.5 mm inner diameter) and bubbled vigorously for 30 min with nitrogen which passed through a mixture of distilled water and DMF (2:9 in the volumetric ratio). With continuing the bubbling, the fluid was irradiated with light from a Xenon lamp (LC-8, Hamamatsu Photonics, Co. Ltd., Hamamatsu, Japan) guided by an optical fiber for 30 min (the distance from the tip of the fiber and the tube was 2 cm). The template was extracted from the particle by suspending in a mixture of 30 mL DMF and 10 mL distilled water and then centrifuged (2600× *g*) for 3 min. Then it was centrifuged in the sequence (a) distilled water, (b) 1 M NaCl at 80 °C, and (c) distilled water; 3 times each for 3 min. The cleaned MIP was dried in a vacuum overnight. The cleaned MIP was dried under vacuum overnight.

#### 2.3.2. Meropenem MIP

The functional monomer MAA (1.39 mmol, 0.12 g), cross-linking monomers MBAA (3.4 mmol, 0.525 g), EDMA (3.4 mmol, 0.675 g), and meropenem trihydrate (0.095 g) were dissolved in 10 mL DMF. To this solution, 0.25 g IG was added and kept in a quartz tube and bubbled with nitrogen gas saturated with DMF for 30 min. The fluid was irradiated with the Xenon lamp for 2 h, by the procedure described in the previous section. The template was extracted from the particle by suspending in a mixture of 30 mL DMF and 10 mL distilled water and then centrifuged (2600× *g*) for 3 min. Then it was centrifuged (2600× *g*) in the sequence (a) distilled water, (b) 1 M NaCl at 80 °C, and (c) distilled water; 3 times each for 3 min. The cleaned MIP was dried in a vacuum overnight, yielding 0.230 g of the MIP-grafted graphite.

#### 2.3.3. Theophylline MIP

The functional monomer MAA (6.69 μmol, 0.57 mg, 0.15 mL) and mixed cross-linking monomers of MBAA (5.8 mmol, 0.9 g) and EDMA (4.5 mmol, 0.9 g) were dissolved in 10 mL DMF. To this, 0.15 g of theophylline was added and mixed thoroughly. Finally, 0.22 g IG was added to this solution and then kept in a quartz tube and bubbled with nitrogen for 30 min to remove any trapped air. The quartz tube was then placed 5 cm before a Xenon lamp for 2 h, by the procedure described in the previous section. After 2 h, the template was extracted using a vacuum filtration method using a glass filter. The suspensions were cleaned one by one using (a) 20 mL DMF, (b) 100 mL 1.5 M NaCl at 80 °C, (c) 100 mL distilled water, and (d) 100 mL methanol. The cleaned MIP was dried using vacuum drying, yielding 0.195 g of the MIP-grafted particles.

#### 2.3.4. Phenobarbital MIP

The functional monomer MAA (6.69 μmol, 0.57 mg, 0.15 mL), cross-linking regulators MBAA (3.8 mmol, 0.6 g) and EDMA (3.0 mmol, 0.6 g) were dissolved in 10 mL DMF. To this, 0.1 g of phenobarbital was added and mixed thoroughly. Finally, 0.16 g IG was added to this solution and then kept in a quartz tube and bubbled with nitrogen for 30 min to purge dissolved oxygen. The quartz tube was then placed 2 cm before a Xenon lamp for 2 h (by the procedure described in the previous section) under continuous stirring and N_2_ bubbling. After two hours, the template was extracted using a vacuum filtration method. The suspension was cleaned one by one using (a) 20 mL DMF, (b) 100 mL 1 M NaCl at 60 °C, (c) 100 mL distilled water, and (d) 100 mL methanol by vacuum filtration. The cleaned MIP was dried using vacuum drying and 0.15 g of the MIP-grafted particles was obtained.

The same process prepared the non-imprinted polymer (NIP) as each MIP except for omitting the addition of each template.

### 2.4. Preparation of MIP-Carbon Paste

Those MIP- or NIP-grafted particles for vancomycin (VCM) and silicone oil (KF-96-300CS, Shin-Etsu Chemical Co., Ltd., Tokyo, Japan) were mixed in a ratio of 7:3 by mass and ground into a paste in a polytetrafluoroethylene mortar and a pestle for about 20 min. In the case of meropenem, theophylline, and phenobarbital, ferrocene was dissolved in the silicone oil (5 mg/mL) as a redox marker before making the paste.

### 2.5. The Ceramic Chip Sensor

**Figure 2:**
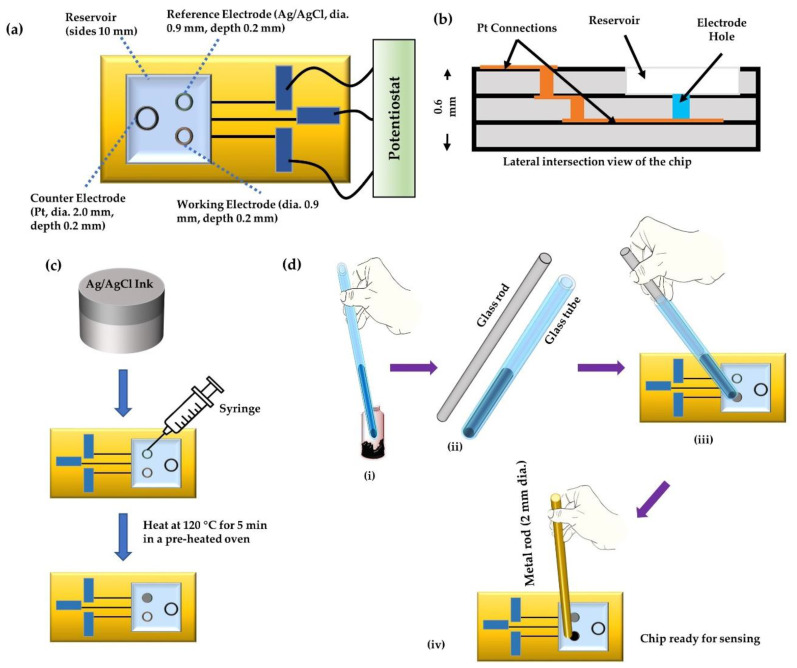
(**a**) Design of the chip along with its dimensions, (**b**) lateral inside view of the chip, (**c**,**d**) illustration of the method of filing the reference electrode and the working electrode.

A ceramic base for the sensor chips (shown in Figure 2a,b) was made from aluminum oxide and platinum wiring. The chip contained three holes for the counter electrode (diameter 2.0 mm), reference electrode (diameter 0.9 mm), and the working electrode (diameter 0.9 mm), each of depth 0.2 mm, connected with platinum wiring at the bottom. The thickness of the chip was 0.6 mm. The chip also comprised of a square sample-reservoir of side 10 mm. The graphite-paste prepared above was used as the working electrode, and Ag/AgCl ink (ALS Co., Ltd. Tokyo, Japan) was used as the reference electrode. The counter electrode was the Pt as in the chip sensor. The Ag/AgCl ink was filled in the reference electrode hole using a syringe (Figure 2c) and heated at around 120 °C for 5 min to make the ink make firm contact with the substrate. To pack the working electrodes, the MIP paste was first packed into the tip of a hematocrit capillary (Hirschmann Labogeräte GmbH & Co. KG, Eberstadt, Germany) with an inner area of 1 mm^2^. A solid glass rod of diameter 1 mm was then pushed inside the other end of the capillary to release the MIP paste into the electrode hole slowly. The filled electrode hole was then tapped with a 2 mm rod to make a compressed working electrode and remove bubbles in the paste at the working electrode, as illustrated in Figure 2d.

### 2.6. The Electrochemical Conditions

The selection of a suitable electrochemical technique is an essential factor in achieving high sensitivity and appropriate limit of detection (LOD) in the quantitative electrochemical analysis. DPV is a voltammetric technique similar to the square wave voltammetry (SWV). In DPV, the potential changes consisting of short pulses are superimposed on a step waveform, which enhances the differentiation of the obtained faradaic currents [29,30]. In fact, in DPV, these short pulses are responsible for the high sensitivity and the differential current helps distinguish the useful faradaic current from any other background processes, especially charging current. Hence, DPV proves to be a better electrochemical analysis tool as compared to the cyclic voltammetry. Therefore, in this study, DPV was selected for the determination of the response of the drugs in the pH 7.4 buffer saline (containing 0.10 M sodium chloride and 0.05 M sodium phosphate) and whole bovine blood.

The electrochemical sensing was carried out using Compactstat.h potentiostat (Ivium Technologies, Eindhoven, the Netherlands), along with IviumSoft version 2.783 [3] (designed to work with Ivium designed electrochemical setup). To get the optimum results, the electrochemical setup was first optimized after several iterations. According to the optimization data, for all the DPV experiments, the parameters were set as follows: initial potential (E_s_) = 0.0 V, terminal potential (E_e_) = 0.9 V, pulse time = 10 ms, pulse amplitude = 50 mV, step potential (E_step_) = 5 mV, scan rate = 10 mV/s and current range set to 10 μA. For each drug, three set of responses were recorded and the average value of the three was used for analysis.

## 3. Results and Discussions

### 3.1. Effect of Ferrocene in the Paste Electrode as a Redox Marker

We have developed a method to detect target chemicals used as a template of MIP through the detection of the faradic current of redox markers (e.g., ferrocyanide) at the MIP-grafted electrode [26,28,31]. This method applies the change in accessibility of redox marker toward the surface of the base electrode coated with the MIP layer by the specific interaction between the target molecule used as a template and the MIP; this can detect even the electrochemically inert targets. However, adding a redox marker into the sample fluid is a troublesome operation. TDM requires a simple operation; thus, an analysis that does not require the addition of an indicator or a marker (reagentless analysis) is mandatory for disposable sensors. We thought the addition of ferrocene, which is oleophilic redox material, in the silicone oil of the paste electrode would work as the redox marker [32]. Ferrocene and its derivatives have been found to: (a) be stable in solution, (b) respond rapidly to many electroactive substances, (c) unreactive with oxygen, (d) require low potential for regeneration, and (e) have fast electron transfer rate. Several other reports have been published previously establishing the role of ferrocene in enhancing the sensing response of a drug sensor [33,34,35,36].

**Figure 3:**
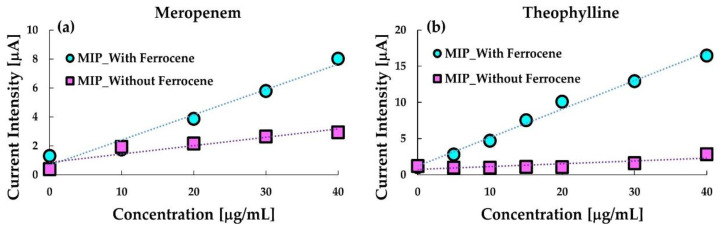
Calibration curves of (**a**) meropenem and (**b**) theophylline with the respective molecularly imprinted polymer (MIP) paste electrodes containing ferrocene in silicone oil (circles) or those without ferrocene (squares).

Figure 3 shows the comparison between calibration curves obtained with and without ferrocene in the MIP-paste electrodes. As indicated in the figure, the ferrocene enhanced the sensitivity of the anodic current at the respective MIP electrodes to meropenem and theophylline. Especially, ferrocene containing the MIP-paste electrode is sensitive to MIP. Further, the anodic current at the ferrocene-free electrode was hardly detected, which indicates that theophylline is electrochemically almost inert at the electrode. These results indicate that the ferrocene dissolved in silicone oil in the MIP-paste electrode can work as a redox marker which enables reagentless sensing of the target. The oxidized ferrocene (ferrocenium cation) produced by the anodic reaction is water-soluble, thus, ferrocene in the electrode may decrease after repeated use. However, the leakage of ferrocene from the electrode would not occur remarkably as far as the electrode is used once only.

Thus, for sensing meropenem, phenobarbital, and theophylline, ferrocene was dissolved in the silicone oil before mixing with the MIP- or NIP- grafted particles in the experiments described hereafter. However, we have already confirmed that reagentless sensing of VCM applies to MIP, including the ferrocenyl group [37,38]; thus, ferrocene was not added in the carbon-paste electrode.

### 3.2. Electroanalysis of the Chip Sensor

**Figure 4:**
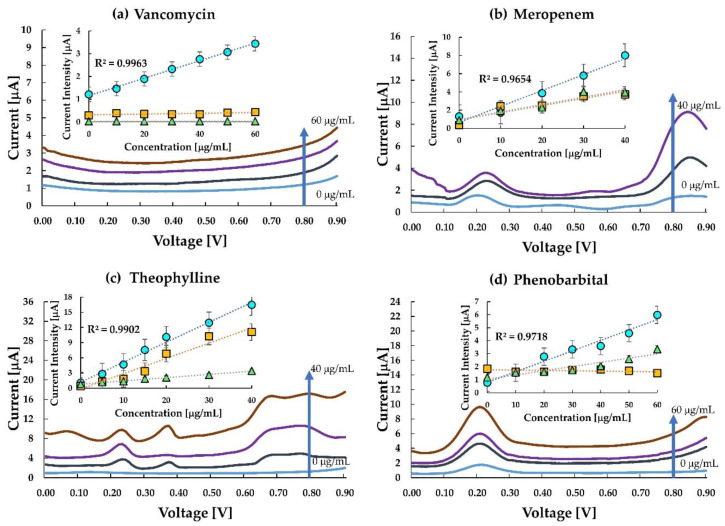
Electroanalysis of the chip sensors for (**a**) vancomycin, (**b**) meropenem, (**c**) theophylline, and (**d**) phenobarbital. The differential pulse voltammograms of the response of MIP to buffer saline in each drug is shown. Insets show the calibration curve for comparison between responses in MIP (circle), non-imprinted polymer (NIP) (square), and untreated carbon paste (triangle) electrodes for each drug. No carbon paste electrodes contained ferrocene.

DPV measurements for all the drugs were performed using the parameters mentioned in Section 2.6. Figure 4a–d shows the voltammogram and calibration curves for the four drugs. The current density obtained at 0.8 V versus Ag/AgCl against the concentration for each MIP, NIP, and untreated carbon-paste (CP) electrodes was sampled with a single use of the sensor chip as shown in the inset of Figure 4a–d. The voltammograms of meropenem, theophylline and phenobarbital have a peak around 0.2 V which represents the redox reactions of ferrocene in the silicon oil used for making the paste electrode. It is quite evident from the calibration curves that the MIP electrodes of the corresponding drugs have more enhanced responses than the NIP and the untreated carbon paste electrodes. The results indicate that interaction between meropenem, phenobarbital, caffeine and the imprinted cavity in the MIP promotes electron transfer from the ferrocene and graphite. The current at the MIP electrode of VCM was sensitive to VCM while those at the NIP-electrode and the untreated CP electrode were insensitive to VCM. It also indicates that VCM is inert at the untreated carbon electrode or the NIP electrode. However, the interaction between the imprinted cavity and the template VCM promotes electron transfer from the ferrocenyl group originated from the ACPF. In the case of VCM, the sensor clearly distinguishes between MIP and NIP.

Similarly, in meropenem, the current responses for NIP and untreated CP electrode were almost the same and relatively lower than the response in the MIP, thereby indicating a higher sensitivity of the meropenem MIP. The almost same response of NIP and untreated CP electrode in comparison to MIP indicated that the imprinted cavities in MIP play an essential role in enhancing the electrode transfer at the anode. Like meropenem, theophylline, too, is seen to be highly responsive to MIP in comparison to NIP. However, the sensitivity of theophylline in the MIP electrode and untreated CP electrode has a small difference only. This may be due to (a) interference of theophylline in the oxidation of ferrocene, and (b) the smaller number of active imprinted cavities created on the MIP surface during the polymerization process. Hence, an improvement in MIP formation may likely improve this response. The difference in response in MIP and untreated carbon paste proves that MIP improves the sensitivity of the electrode. Since the MIP has impregnated sites for test molecules, it gets more accessible for the test molecules to get attached to the MIP. This reduces the resistance on the surface, thus increasing the current density.

In the case of phenobarbital, the response in MIP, NIP and untreated CP electrode is almost overlapping in the lower concentration range, and slowly differentiating as the concentration increases. One of the reasons could be the smaller number of molecules interacting with the available drug cavities resulting in lesser current transport. At higher concentration ranges (≥20 μgmL^−1^), MIP shows a distinctive response. The response curve in the untreated CP electrode is almost flat, indicating a negligible change in current at the electrode with a change in concentration of the analyte. As the concentration increases, a greater number of phenobarbital molecules are available to occupy the imprinted cavities of the MIP, thereby increasing the response current.

### 3.3. The Reproducibility and Selectivity

**Figure 5:**
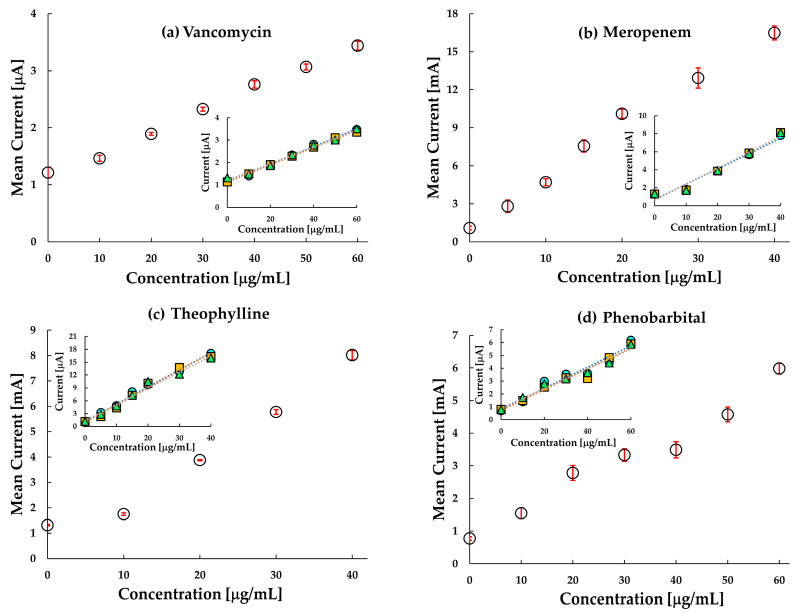
Reproducibility test in saline of the target by MIP for: (**a**) vancomycin, (**b**) meropenem, (**c**) theophylline and (**d**) phenobarbital. The mean current value for three sensors at each concentration is plotted with standard deviation at each value as the error. The response at every concentration for three different chips for every drug is shown in the inset.

The development of a reproducible sensor is one of the major challenges faced in the process of electrochemical sensor fabrication. However, by using the sensors only once, i.e., disposable sensor, the performance can be substantially improved. As an attempt to improve the reproducibility of the sensor, our sensor has been used only once for a single concentration and the reproducibility of electrodes at each concentration is quite good with the standard deviations less than 1 in all the cases. The reproducibility at each concentration was measured by three electrodes at each concentration for each drug. The reproducibility data of the electrodes are plotted in Figure 5. Coefficient of variance (CV) was calculated for each concentration of each drug. For the VCM sensor, the variability of zero concentration was around 0.07% while for 60 μg/mL was around 0.22%. In meropenem, the CV was around 0.2% for all the data. For the theophylline sensor, the CV for zero concentration was around 0.13% while for 60 μg/mL was around 0.03%. For the phenobarbital sensor, the variability of zero concentration was around 0.7% while for 60 μg/mL was around 0.03%. All these data indicate that every set of measurement for a particular concentration with a different chip had a response value close to the mean value at each concentration. However, it is worth noting here that in all the sensors, as the concentration increased, the CV value decreased, i.e., the closeness of the response to the mean value increased. Thereby, we can say that since the variability of the data around every concentration for each drug was well in the range, and hence, the chip sensor can obtain reproducible current responses at every concentration for each drug. Further, as the measurement errors are small, it is very likely that the sensor is reliable.

**Figure 6:**
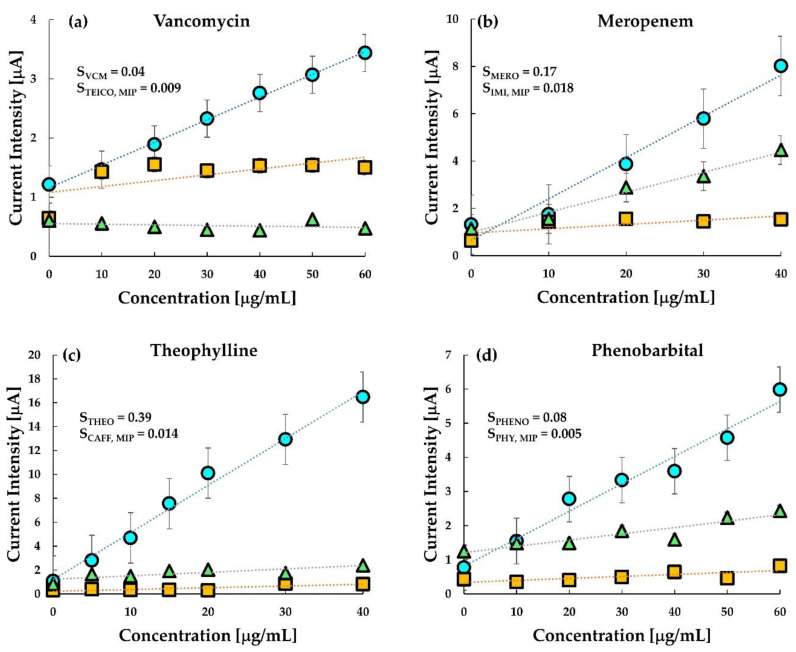
Selectivity test in saline of the target by MIP (circles), the analogue by MIP (squares) and analogue by untreated carbon paste (triangles): (**a**) vancomycin, (**b**) meropenem, (**c**) theophylline and (**d**) phenobarbital.

The development of sensing electrodes with a high affinity of the binding sites for the target drug and low affinity for a structurally analogous drug is expected to result in more effective drug monitoring. Hence, selectivity is a critical pharmacodynamic parameter to be addressed for effective therapeutic monitoring. The selectivity of the molecularly imprinted sensors is chiefly governed by the imprinting reactions [39]. In this regard, the selectivity of the prepared MIP sensors was tested with drugs that have a similar structure to that of the target drug (Table 1), along with the test on untreated CP electrodes. Figure 6 shows the selectivity study of the chip sensor. From the figure, it is clear that the selectivity of the sensors was excellent and comparable even in the presence of a drug similar to the target analyte. The selectivity of target drugs is higher than the other drug both in MIP and in untreated carbon paste. Vancomycin MIP is seen to be slightly selective towards teicoplanin, however, the sensitivity was more than five times in the presence of VCM than in teicoplanin. The response of the untreated CP electrode towards teicoplanin was, however, slightly greater than vancomycin as observed previously (Figure 4). This result indicates that selective monitoring of VCM can be done using the fabricated MIP sensor.

In the case of Meropenem, it was interesting to note that the response of untreated CP electrodes towards its analogue drug imipenem was almost the same as its response in meropenem. Interestingly, the MIP sensor showed an excellent selectivity towards imipenem with an approximately ten times higher sensitivity value. A similar selectivity was observed in theophylline MIP against its off-target drug caffeine. MIP sensor of theophylline was insensitive to caffeine. However, the untreated carbon paste electrode was also sensitive to theophylline but insensitive to caffeine. The observed selectivity indicates that theophylline has higher electrochemical reactivity than caffeine at the surface of the graphite anode but does not indicate the specificity of the MIP of theophylline.

Further, the selectivity of the MIP sensor was higher for phenobarbital than its analogue, phenytoin. However, sensitivity towards phenytoin was quite prominent in the untreated CP electrode. Nevertheless, it may be noted here, that the similarity in response towards phenobarbital and phenytoin is very high. This indicates that although the untreated CP electrode is responsive towards both the drugs, the selectivity is almost negligible. The sensitivity calculated for MIP in phenobarbital was 0.08, while that in phenytoin was only 0.005, indicating not only a good selectivity but also the higher sensitivity of the developed MIP sensor.

### 3.4. Analysis in Bovine Whole Blood

**Figure 7:**
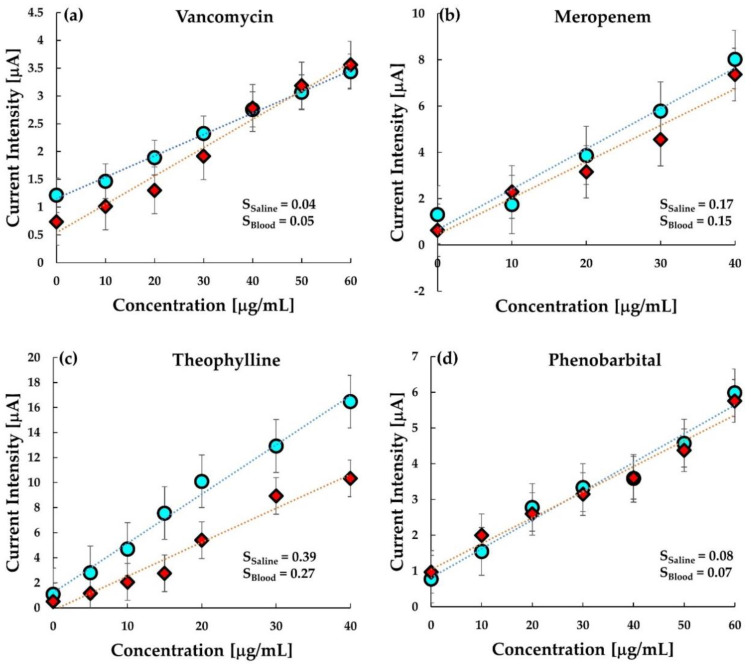
The comparison of MIP sensor response in saline and in blood for (**a**) vancomycin, (**b**) meropenem, (**c**) theophylline, and (**d**) phenobarbital. Circle (○) represents response in saline, and rhombus (◇) represents response in whole bovine blood.

As shown in Figure 7a–d, a comparative analysis of MIP in buffer saline and whole bovine blood has been performed. Interestingly, the graph clearly illustrates the similarity between the response of MIP in buffer saline and blood. The sensitivity in both buffer and bovine blood is quite comparable. This indicates that the MIP sensor response is stable and reproducible in both buffer saline and whole blood.

However, in the case of theophylline, the response in the blood is relatively less than the response in the buffer. The ratio of binding of theophylline with serum protein (40%) is not significantly larger than that of VCM (34%) or phenobarbital (20–45%); thus, it is unlikely that the protein binding decreases the sensitivity in blood [40,41,42,43,44,45]. The lower sensitivity to theophylline is probably due to the interaction of some coexistence in blood resulted from the insufficient specificity of the MIP comparing with other MIPs is this work. The dynamic range at the MIP electrode versus the concentration of VCM and phenobarbital is seen in the range of 0–60 μgmL^−1^, which covers the therapeutically effective concentration level of vancomycin and phenobarbital in plasma varying from 20 to 40 μgmL^−1^ and 15 to 30 μgmL^−1^, respectively. Similarly, the dynamic range in meropenem and theophylline and is seen in the range of 0–40 μgmL^−1^ corresponding to the respective therapeutic ranges (Table 2).

Further, the response curves for all the drugs were fitted by linear regression, and each of them showed an R-squared (R^2^) value greater than 0.900. This owes to the uniformity of the working electrode of the sensor chip fabricated after thorough homogenization of the MIP-grafted carbon paste. Looking at the responses, it can be concluded that the current intensity changes quite linearly with the change in concentration of the analyte drug. Further, the slope of the linear fit line was calculated to determine the sensitivity of the MIP sensors in the target drug in the drug with a similar structure. The sensitivity of each drug determined from the linear-fit line slope has been tabulated in Table 3. The limit of detection (LOD) was calculated using the formula [49]:

(1) $$\mathrm{LOD}=3.3\times \;\frac{SD_{r}}{S}$$

where $SD_{r}$ is the standard error of the linear regression, and S is the slope obtained from the equation of the linear fit line. For most of the drugs, the LOD was quite low, except for theophylline, the reason is probably the insufficient MIP formation. However, each LOD is smaller than the minimum therapeutic window of the respective drug in plasma. Thus, the sensitivity of the sensor using MIP is sufficient to determine whether the drug level is sufficient or not.

### 3.5. Possibility of “Disposable” Sensor for TDM

The results obtained for the MIP-based sensor for all the four drugs are entirely satisfactory. Not only the sensitivity but also the selectivity of the sensors is good. The high selectivity and reduced analysis time add to the advantage of this sensor. The main aim of the present sensor is to use it in therapeutic drug monitoring of various drugs that are critical for the treatment of specific body conditions, including anti-microbial drug resistance, epilepsy, and seizure. VCM is a well-used drug against infection of Gram-positive bacteria, especially the first-choice drug for the treatment of methicillin-resistant Staphylococcus aureus, and its TDM is quite essential to reduce the risk of kidney failure or nephrotoxicity [46].

Meropenem is an important anti-Gram-negative bacteria drug that is often used in the treatment of infections of the abdomen, and the skin. Thus, the TDM of meropenem is highly recommended for patients to avoid a higher risk of kidney failure [11,14] and prevention of the creation of the resistant bacteria which has been spreading recently [50,51,52,53]. Theophylline is also a crucial drug widely used for the treatment of chronic lung diseases such as asthma and chronic obstructive pulmonary disease. However, an overdose of theophylline may fail the central nervous system, and therefore its TDM is required [48]. Likewise, phenobarbital is an anticonvulsant and is also recommended to be monitored as its use is quite prevalent in developing countries for the treatment of epilepsy [54]. Keeping in view the prevalent use of these drugs in poor and developing countries, the ceramic chip sensor with the MIP-grafted carbon-paste electrode is a cheap alternative to the expensive chromatography or immunoassay analyses available for TDM. Notably, in the developing countries of Asia and Africa, where TDM is often skipped due to the enormous expenses for the analysis, this simply operable ceramic chip sensor can be an economical alternative.

The ceramic chip has an active electrode area of around 0.64 mm^2^. This active area of the electrode, along with the active binding sites in the MIPs, plays a vital role in the current sensitivity of the MIP sensors. As can be seen from Figure 4, the MIPs for each drug show a better response than the untreated carbon paste. Although the exact sensing mechanism of these sensors cannot be elucidated at this stage, it can be related to some of the previously established theories of sensing. The underlying mechanism of electrochemical sensing is based on the generation of signals due to interference of specific interaction between the template and the imprinted cavity in the MIP toward electron transportation the redox marker and the working electrode. Under this process, with an increasing concentration of analytes, further rebinding may induce a stronger redox reaction and increase peak current value. Additionally, the shape of the imprinted cavities complements that of the prototype following the lock-key principle [55]; thus, when a target with the same configuration as that of the template comes into the vicinity of the MIP, it immediately responds to the analyte. This mechanism can be the reason for higher selectivity in MIP sensors.

It is interesting to note that the chip sensors show a similar response in bovine blood (Figure 7) as in buffer saline solution. Although a better response can be expected when used in plasma alone, it is almost satisfactory at this stage to have responses similar to that in the saline. In a real-time situation, it is easier to use disposable sensors to reduce the contamination and infection risks of operators and also to reduce the cost of the analysis. Not only this, but the turnaround time for most of the conventional TDM techniques is also 1–2 h because they require troublesome operations, e.g., removal of blood cells or proteins. The present ceramic chip sensor, on the contrary, by the use of differential pulse voltammetry, provides results in less than two minutes and, therefore, can be offered “bed-side TDM” where physicians and pharmacist can decide the optimal timing and amount of drug administration based on the real-time data of drug concentration. Hence, this “single-use” sensor can be implemented for the therapeutic monitoring of these critical drugs by not only offering an easy-to-use and bed-side analysis method but also reducing the cost of analysis as the over-all cost of the ceramic chip sensor is less than USD 5.

## 4. Conclusions

In this research, electrochemical sensors based on MIP-modified carbon-paste electrodes on a ceramic chip for selective and sensitive determination of concentrations of four significant drugs: vancomycin, meropenem, theophylline, and phenobarbital, were developed. MIP in all the cases proved to be a highly selective recognition element in the modified carbon-paste electrode configuration. The presented sensor has excellent reproducibility, ease of preparation and regeneration, suitable response time, low cost, and simplicity. Additionally, the presence of interfering species had a minimal effect on the sensor’s analytical response when measured in bovine blood. This method can be used for therapeutic analyses in local hospitals as “bed-side TDM” by considering the successful application of the proposed method for drug determination in pharmaceutical formulations and human blood samples.

## Figures and Tables

**Table 1:** Structural similarity between target drugs under study and their analogues.

Target		Analogue of the Target	
Vancomycin	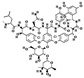	Teicoplanin	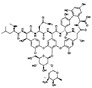
Meropenem	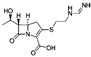	Imipenem	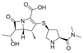
Theophylline	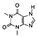	Caffeine	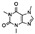
Phenobarbital	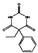	Phenytoin	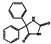

**Table 2:** Therapeutic range of various drugs.

Drug	Therapeutic Window in Plasma	Reference
Vancomycin	20–40 μg mL^−1^	[46,47]
Meropenem	14–26 μg mL^−1^	[5,11]
Theophylline	5–15 μg mL^−1^	[7,48]
Phenobarbital	15–30 μg mL^−1^	[9,10]

**Table 3:** Sensitivity and limit of detection (LOD) of MIP, NIP, and untreated electrode towards various drugs.

Drug	Sensitivity(mA·L/g)				LOD (μg/mL)	
	MIP (Saline)	NIP (Saline)	Untreated (Saline)	MIP (Blood)	MIP (Saline)	MIP (Blood)
Vancomycin	0.04	0.001	0.000	0.05	2.51	7.47
Meropenem	0.17	0.078	0.082	0.16	4.04	4.76
Theophylline	0.39	0.302	0.064	0.27	2.39	3.53
Phenobarbital	0.08	0.002	0.032	0.07	1.37	4.91
